# The circadian clock participates in seasonal growth in Norway spruce (*Picea abies*)

**DOI:** 10.1093/treephys/tpae139

**Published:** 2024-11-03

**Authors:** David Lázaro-Gimeno, Camilla Ferrari, Nico Delhomme, Mikael Johansson, Johan Sjölander, Rajesh Kumar Singh, Marek Mutwil, Maria E Eriksson

**Affiliations:** Department of Plant Physiology, Umeå Plant Science Centre, Umeå University, Umeå SE-901 87, Sweden; Max-Planck Institute for Molecular Plant Physiology, Am Muehlenberg 1, Potsdam, 14476, Germany; Department of Forest Genetics and Plant Physiology, Umeå Plant Science Centre, Swedish University of Agricultural Sciences, Umeå SE-901 83, Sweden; Department of Plant Physiology, Umeå Plant Science Centre, Umeå University, Umeå SE-901 87, Sweden; Department of Plant Physiology, Umeå Plant Science Centre, Umeå University, Umeå SE-901 87, Sweden; Department of Plant Physiology, Umeå Plant Science Centre, Umeå University, Umeå SE-901 87, Sweden; Max-Planck Institute for Molecular Plant Physiology, Am Muehlenberg 1, Potsdam, 14476, Germany; Department of Plant Physiology, Umeå Plant Science Centre, Umeå University, Umeå SE-901 87, Sweden

**Keywords:** bud burst, delayed fluorescence, gene regulatory network, rhythmicity

## Abstract

The boreal forest ecosystems of the northern hemisphere are dominated by conifers, of which Norway spruce (*Picea abies* [L.] H. Karst.) is one of the most common species. Due to its economic interest to the agroforestry industry, as well as its ecological significance, it is important to understand seasonal growth and biomass production in Norway spruce. Solid evidence that the circadian clock regulates growth in conifers has proved elusive, however, resulting in significant gaps in our knowledge of clock function in these trees. Here, we reassess the impact of the circadian clock on growth in Norway spruce. Using a combination of approaches monitoring the physiology of vegetative growth, transcriptomics and bioinformatics, we determined that the clock could be playing a decisive role in enabling growth, acting in specific developmental processes influenced by season and geographical location to guide bud burst and growth. Thus, the evidence indicates that there is time for spruce.

## Introduction

Boreal forests are dominated by coniferous trees (Shorohova et al. 2011), such as Norway spruce (*Picea abies* [L.] H. Karst.). These species are highly adapted to local conditions and natural populations show geographical clines in physiological traits such as bud set and bud burst from north to south (Langlet 1960, Worrall and Mergen 1967, Beuker 1994). Photoperiodic control of bud set, involving circadian time keeping, is firmly established (Vince-Prue et al. 2001). Typically, these habitats provide only a short period of permissive environmental conditions during which trees must complete their biological functions, including seasonal growth and reproduction, and a far longer period during which they must resist the cold, while the trees are dormant.

A circadian (circa 24 h) clock has evolved in most organisms to synchronize their growth and align development with the environmental cycles experienced on Earth. These clocks respond to external stimuli with light (day length) and temperature being the principal ones (Salomé and McClung 2005). The circadian clock of angiosperms regulates many aspects of plant growth and development, ensuring metabolism and physiology align with local conditions. In trees such as sweet chestnut (*Castenea sativa*) (Ramos et al. 2005) and aspen (*Populus* sp.) (Takata et al. 2009, Ibáñez et al. 2010, Filichkin et al. 2011), daily growth and coordination of stress adaptation during periods of active growth depends on the proper functioning of circadian clock components such as *LATE ELONGATED HYPOCOTYL 1* (*LHY1*), *LHY2*, *TIMING OF CAB2 EXPRESSION 1* (*TOC1*) and *ZEITLUPE* (*ZTL*) (Edwards et al. 2018, Jurca et al. 2022). In *Populus* sp., a functional clock sets the responses to the photoperiodic rhythms in the environment, ensuring that the physiological changes necessary for growth cessation and bud set occur in a timely fashion (Ibáñez et al. 2010), and this trait show association with variation in circadian genes in natural populations (Ma et al. 2010)*.* Noteworthy, the timing of bud burst, which was considered mainly to be affected by spring temperatures and water availability, is under control of the circadian system (Ibáñez et al. 2010). Natural variation in light input pathways, as well as in the circadian clock mechanism, of crop species such as barley, tomato and rice has enabled selective breeding programs to develop varieties that are productive at higher latitudes (Faure et al. 2012, Yang et al. 2013, Müller et al. 2016, 2018).

Several studies have shown the clock system in *Populus* sp. resembles that of Arabidopsis (*Arabidopsis thaliana*), a model angiosperm (Ibáñez et al. 2010, Ramos-Sanchez et al. 2019, Jurca et al. 2022, reviewed by Singh et al. 2021). Although the evolution of the circadian clock pre-dates the division between gymnosperms and angiosperms, which occurred around 400 million years ago, the full details of how the circadian clock system functions in gymnosperms such as conifers remains unclear. Norway spruce has been investigated at the molecular level to determine whether it possesses a circadian system. Reports of reductions in the expression of putative clock genes and observations that delayed fluorescence (DF) of photosystem II (PSII) lacked periodicity (Gyllenstrand et al. 2014) and did not support clock function during active growth. For the last decade, therefore, it has been widely considered that conifer growth is not regulated directly by the circadian clock, a conclusion that required re-evaluation of the status of circadian clock genes (Nakamichi 2011). Recent studies (Nose and Watanabe 2014, Ferrari et al. 2019, Scott et al. 2020, Michael 2022), however, have cast doubt on this conclusion. According to the information we have available, we are proposing the following hypothesis:

- The developmental stage of Norway spruce is essential for the study of the circadian clock and rhythmicity. Bud burst is an important aspect in tree phenology being under-explored.
- We want to unravel the role of light and temperature on gymnosperms plant growth in natural conditions (no training) and how all of this environmental factors affects the circadian rhythm.

## Materials and methods

### Plant material

Forty-eight grafted plants (clones) from the Norway spruce (*Picea abies* [L.] H. Karst.) natural community clones S23K8720483 (otherwise known as clone 483) from the Näskott S population (Lat. 63.27° N; Long. 14.25° E) (*n* = 35) and S23K8720142 (otherwise known as clone 142) from the Näverbet population (Lat. 66.7° N; Long. 22.53° E) (*n* = 13), were obtained from Skogforsk, Sävar, Sweden, in 2019. The root stock used for grafting the clones come from seed plantation Domsjöänget 130 in 2017.

### Growth conditions

Clones were grown in the Wallenberg greenhouse at UPSC, Umeå (Lat. 63.82° N, Long. 20.26° E), Sweden, under natural conditions and transferred indoors, between October and May (winter–spring), to 80% humidity and photoperiods light (L): dark (D) 8 h:16 h at 6 °C:6 °C (short days LD 8:16, cold conditions) with light fluency of 200 μmol m^−2^ s^−1^ (Osram Powerstar HQI-T 400 W/D lamps, Osram). Before bud burst between 27 and 30 May, clones were moved outdoors to natural conditions of light and temperature.

Plants were potted in 7 L pots with fertilized peat:perlite mix 4:1, watered and fertilized with SW Bouyant Rika S by greenhouse staff out of the experimental frames.

### Bud burst, DF studies

**Figure 1:**
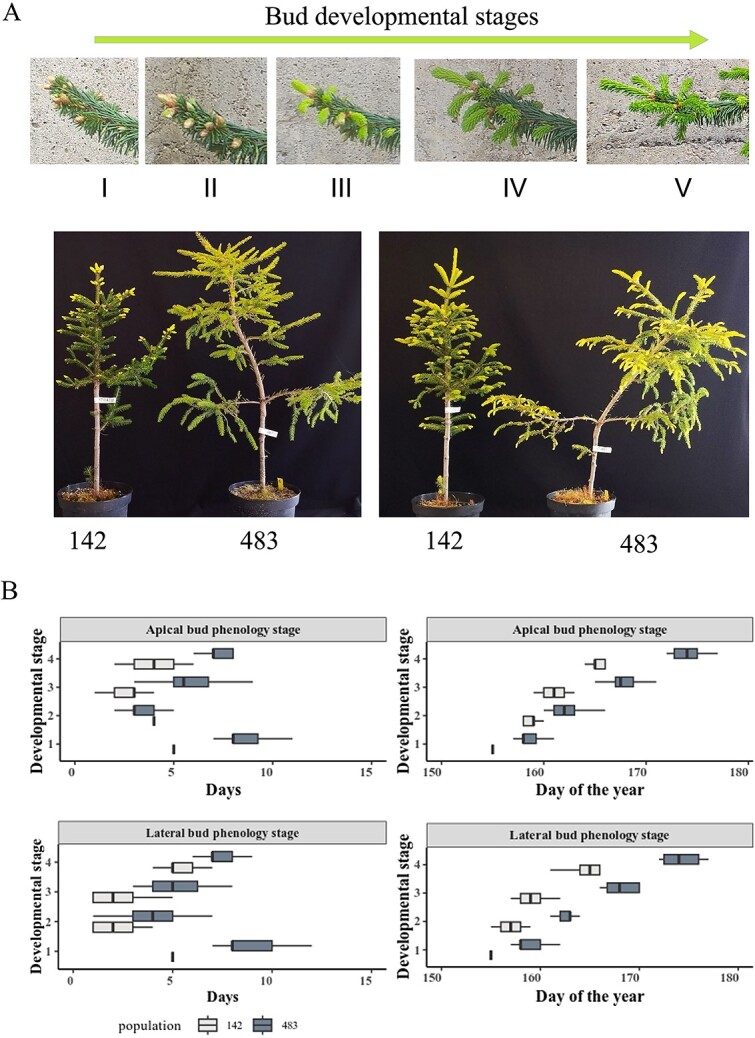
Norway spruce clones 142 and 483 grown in the field under natural conditions differ in their timing of springtime phenology and show partially rhythmic behavior during active growth. (A) Reference developmental stages selected from one representative along time from clone 142 (upper panel): Stage I 2022/05/30, Stage II 2022/06/03, Stage III (2022/06/07), Stage IV (2022/06/12), Stage V (2022/06/20). Picture showing in detail the differences in bud burst between the two clones in the same day (lower panel): 2023/05/23 (left), 2023/06/05 (right). (B) Assessment of bud burst phenology of apical and lateral buds. Data from both clones are displayed. Left-hand panel: absolute number of days to complete each developmental stage; right-hand panel: relative development across days of the year. The two clones differed significantly. Clone 142, which originated above the Arctic circle, completed bud burst faster and within a shorter period. ANOVA apical buds *P*-value < 2e−16, ^***^; ANOVA lateral buds *P*-value 1.93e−10, ^***^.

Photographs of 48 individuals from the two natural populations were taken daily from 27 May 2022 to 27 June 2022, to record bud burst development and to classify each stage of apical and lateral bud development according to the Ducci classification (Ducci 2012). Figure 1A summarizes plant 142_44 on 30 May, and 3, 7, 12 and 20 June as evidence of the differential developmental stages.

For DF analysis, whole bud samples were collected from both populations on 15 June 2022 (week 24), when the first buds from population 142 had reached developmental stage III. A further 20 bud samples were collected on 4 July 2022 (week 27), when buds from population 483 had reached developmental stage III and buds from population 142 had reached developmental stage IV, in order to perform a DF analysis under the local and seasonal light conditions (Figure S1 available as Supplementary data at *Tree Physiology* Online). Data obtained using a in-house Shiny app (https://github.com/dlazarogi/Combined-Climate-Data-Analysis).

A second in-depth DF analysis was performed on populations 142 and 483 between 25 May (week 21) and 16 July (week 28) 2023, when they were in developmental stages II to IV; samples were collected from trees grown under near-constant natural light.

Samples were surface-sterilized with ethanol 70% (2 changes 1 min), rinsed with Milli-Q water (1 min) and placed on square plates (12 × 12 cm) containing half-strength ½ MS (Murashige and Skoog 1962) medium without sucrose, under LL at a constant temperature of 22 °C. The light consisted of equal parts blue (470 nm) and red light (660 nm) from 20 μmol m^−2^ s^−1^ light-emitting diodes (MD Electronics, Warwick, UK). Rhythms of DF were recorded following lights-off using a Photek HRPCS 5 photon counting camera (Photek Limited, St Leonards on Sea, UK). Photek Image 32 v 6.0.3, with 1 min exposure, loop time 60 min, initial delay 0 s and binary slice, was used to register the images and measure the regions of interest. The background subtraction was equally applied to all images in all experiments and every individual matrix result from each experiment was linear de-trended and normalized (Gould et al. 2006). Periodicity was calculated using Biodare2 (https://biodare2.ed.ac.uk) using fast Fourier-transformed non-linear least-square analysis (FFT NLS) (Zielinski et al. 2014), considering Circadian Time (CT) 0 at subjective dawn with a minimum 72 h and a maximum 125 h window across DF experiments.

DF burst data were and normalized (−1 and 1 interval) using the following Python code:

import numpy as np.

from sklearn.preprocessing import MinMaxScaler.

# Replace file.txt with source,1 data per line.

data_file = "file.txt”.

data = np.loadtxt(data_file).

# Normalize data.

data_reshape = data.reshape(−1, 1).

scaler = MinMaxScaler().

normalized_data = scaler.fit_transform(data_reshape).

# Calculate trend.

x = np.arange(len(normalized_data.flatten())).

coeff = np.polyfit(x, normalized_data.flatten(), 1).

trend = np.polyval(coeff, x).

# Detrend data.

detrended_data = normalized_data.flatten()—trend.

print(‘Normalized detrended data: \n’,'\n'.join(str(i) for i in detrended_data)).

### Infrared gas analysis of photosynthesis and gas exchange

Clones from populations 142 and 483 were monitored during the period of bud burst when they reached developmental level four using a Li-COR 6400 infrared gas analyzer (IRGA)-based gas exchange (Gs) portable analyzer system (Li-Cor Inc., Lincoln, NE, USA) installed with an Opaque Conifer iChamber (part 6400-22) that controlled irradiance (1000 mmol photons m^−2^ s^−1^), temperature (25 °C), CO_2_ concentration (400 mmol mol^−1^), relative humidity (40%) and flow rate (500 cm^3^ min^−1^). Measurements were made at CT 6 (i.e., 6 h after dawn) of net CO_2_ assimilation rate (A_n_), stomata conductance (g_n_), the internal CO_2_ of the needle (Ci) and the transpiration rate, with five replicates per population and three measurements per individual. Higher times exceeded the frame to measure under the same timeframe more individuals for each replicate, due to the necessity to stabilize the device between measurements.

### RNA-seq sampling, analysis and gene regulatory network

Fifty-two rooting cutting trees from natural community clone S23K8720483 were provided from Skogforsk, Sävar. Controlled diurnal and circadian experiments were conducted: LD, 18 h:6 h at 18 °C:18 °C (long days LD 18:6), which represent the conditions at the geographic origin of the population (Ekberg et al. 1979) and LL, 24 h:0 h at 18 °C:18 °C (continuous light, LL) collecting complete buds samples every 4 h. The period between LD and LL experiment included an exposure for 72 h of constant light to retrain and reset the conditions employed in the first part of the experiment. Samples were flash-frozen in liquid nitrogen and stored at −80 °C. Total RNA from needles was extracted using the CTAB method (Chang et al. 1993), transferred to RNeasy extraction columns (Qiagen GmbH, Hilden, Germany) for further purification and on-column DNAse treatment. An RNA-seq library was constructed using Illumina Total RNA Library Prep Kit with Ribo-Zero Gold (Illumina, Inc., Cambridge, UK) and analyzed by Bioanalyzer (Agilent Technologies) at SciLifelab, Stockholm, Sweden.

Sequence reads pre-processing and quality assessment of the raw data were performed according to the standard guidelines, using FASTQC_0.10.1, SortmeRNA-2.0 to filter RNA contaminants (Kopylova et al. 2012). Trimmomatic-0.022 (Bolger et al. 2014) and STAR-2.4 were used for reading alignments (Dobin et al. 2013).

Expression values were imported into R (v3.3.0; R Core Team 2015) using Bioconductor v3.3 (Gentleman et al. 2004). For data quality assessment (QA) and visualization, read counts were normalized using variance stabilized counts (VST), as implemented in the Bioconductor DESeq2 package v1.12.0 (Love et al. 2014). The biological relevance of the data—e.g., similarity between biological replicates—was assessed by principal component analysis (PCA) and other visualizations (including heatmaps) using custom R scripts. Hierarchical clustering was performed using the Pvclust R package (Nakano et al. 2006).

Rhythmic genes were identified by using the JTK algorithm (Hughes et al. 2010). To validate the results obtained by JTK, we used the Haystack algorithm (Mockler et al. 2007, Michael et al. 2008). The correlation cut-off was set to 0.8, the fold-change to 1, the background cut-off to 1, and the *P*-value cut-off to 0.05.

Read count per transcript was also checked using Salmon v0.11.2 (Patro et al. 2017) for gene meta-network inference. The abundance values were imported using the Bioconductor tximport package v.1.11.6 (Soneson et al. 2015), and normalized by light condition (LD, LL) independently using a variance stabilizing transformation (VST), with the sampling time according to design. The two datasets were filtered for low VST expression values (average biological replicates ≤1 in all sampling times).

The resulting matrices were used as input to generate gene inference meta-networks using Seidr (Schiffthaler et al. 2023). Briefly, 11 gene network inference methods were run (ARACNE, CLR, el-ensemble, GENIE3, llr-ensemble, NARROMI, Partial Correlation, Pearson, PLSNET, Spearman, and TIGRESS), then aggregated using the Inverse Rank Product (IRP). The resulting fully dense network (every gene has a link to all other genes) was then filtered using a backbone approach with a 1% cut-off (for full methods, see (Schiffthaler et al. 2023). The network’s validity was assessed using a positive edges gold standard derived from KEGG pathways, generating AUROC values (area under the receiver operating characteristic curve). Both aggregated backboned networks had an AUC of 0.676.

The first-degree neighborhood of both the LD and LL data were retrieved and merged into a single network using the R (v4.2.1; R Core Team 2020) igraph (Csárdi et al. 2016) package, keeping the origin of the edge (LD only, LL only or both) as an attribute. This network was further visualized and processed using Cytoscape (Shannon et al. 2003).

The *Picea abies* S23K8720483 transcriptome RNA sequence data were deposited in EMBL's European Bioinformatics Institute as PRJEB8220.

### Statistical analyses

Physiological data were analyzed with R version 3.6.3 and Rstudio version 1.2.5033. Statistical analyses included outlier detection, Shapiro–Wilk test for normality of the data, analysis of variance (ANOVA) and Games–Howel as post hoc if data were statistically significant.

Bud burst analysis was evaluated using Shapiro–Wilk test for normality, ANOVA for linear model where the value was the number of days each plant needed to achieve the specific developmental stage and the independent variable was the developmental stage. Games–Howell as post-hoc. Representative results are shown in Figure 1B and Games–Howel post hoc test in Table 1.

**Table 1:** Analysis of variance test result and Games–Howell *post hoc* analysis comparison for apical and lateral timing to achieve scores in buds burst. Group 1 (*n* = 13), group 2 (*n* = 35).

Group 1	Group 2	Estimate	Conf. low	Conf. high	*P* adj.	*P* adj. signif.
142 A_score I	483 A_score I	3.2967033	1.8789052	4.714501	0.0000005	^****^
142 A_score II	483 A_score II	2.729443	0.7800908	4.678795	0.002	^**^
142 A_score III	483 A_score III	5.9761273	3.4682334	8.484021	0.0000001	^****^
142 A_score IV	483 A_score IV	6.5045249	2.486539	10.522511	0.0002	^***^
142 L_score I	483 L_score I	3.5769231	1.9958784	5.1579678	0.0000013	^****^
142 L_score II	483 L_score II	5.1923077	3.1697136	7.2149017	0	^****^
142 L_score III	483 L_score III	7.8433048	4.5876359	11.0989737	0.0000001	^****^
142 L_score IV	483 L_score IV	6.8197115	2.0535981	11.585825	0.001	^***^

^*^^*^: Significant at the 1% level; ^*^^*^^*^: Significant at the 0.1% level; ^*^^*^^*^^*^: Highly significant.

The DF results were import the values in Biodare2 and analyzed by their internal tools. Threshold was set in a *P*-value ≤0.05. Data generated in the DF experiments are available from Biodare2: 27,508 (142 June 2022), 27,579 (483 Stage II arrhytmic 2022), 27,511 (142 July 2022), 27,249 (142 weeks analyzed 2023), 27,254 (483 weeks analyzed 2023) and 28,466 (April with dormant buds last year needles).

## Results

### Rythmicity analysis during bud development

We wanted to evaluate whether developmental differences between apical and lateral buds during the growth season exist. Our results revealed that the clone 142 initiated each developmental stage significantly earlier and showed faster progression than the clone 483 (Figure 1B**,**  Table 1). The clone 483 reaches the same bud developmental stage as clone 142 between 7 and 16 days later. This fact affected bud sampling. For the same week of the year of the experiment, the developmental stage of clone 483 was different than clone 142.

**Figure 2:**
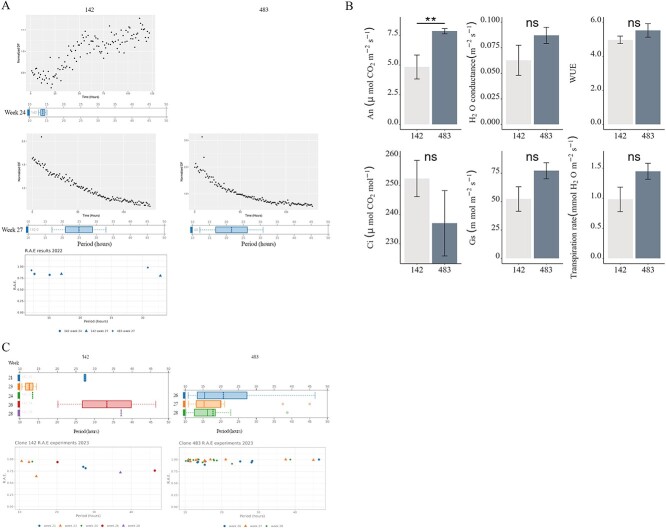
Delayed fluorescence (DF) and relative amplitude error (RAE) results from photosystem II and photosynthesis of Norway spruce increase mid- bud growth and display variable rhythms across developmental stages and between clones. (A) Delayed fluorescence (DF) and RAE from buds from clones 142 and 438 at developmental stages III and IV sampled at the weeks indicated during spring 2022. The DF data from each genotype shown in each chart were normalized to mean but not detrended. Period estimates were obtained using detrended, normalized data from these samples and are shown as box plots below the charts; only the replicates that passed Biodare2 test of FFT NLS rhythms between assayed LL time-window are included (see Table 2 for an overview of DF assays). (B) Measurements in photosynthetic activity in stage IV buds. Graphs are comparing clones 142 and 483 for: photosynthesis (An), conductance (Ci), Gs, water conductance, water-use efficiency (WUE) and transpiration. Significant differences are indicated with ^*^; ns: non-significant results; *n* = 5. ANOVA and games Howell as post-hoc (*P*-value = 0.05). (C) Delayed fluorescence (DF) and RAE results from buds at developmental stages III and stage IV sampled at the weeks indicated during spring 2023 from clones 142 and 483. Period estimates were obtained using detrended, normalized data from samples; box plots are shown for replicates that passed Biodare2 test FFT NLS. Relative amplitude error (RAE) calculated from results for each experiment.

We performed DF analysis in 2022 and 2023. For the first analysis in June 2022, clone 142 displayed rhythmicity. The level of DF increased over time in this clone and period analysis revealed an ultradian period of around 14 h. Buds were at developmental stage III (Figure 2A, upper left panel). When we repeated the DF analysis in July 2022, we observed rhythmicity in buds from both clones, although the amplitude of DF expression reduced over time (dampened) (Figure 2A, lower panels). The average period in late stage III buds from clone 142 was around 24.92 h (Figure 2A, lower left panel), whereas it was 21.51 h for stage III buds from clone 483 (Figure 2A, lower right panel). The period estimates are shown in Table 2. These results suggested there may be a limited window during which bud development is rhythmic, and probably the circadian clock is involved, loosing rythmicity as the buds go to fully developed needles. Based on these results, we repeated the DF measurements in 2023, increasing the number of time points of study. We focused on the window between stages II, III and IV of bud burst (Figure 2C). We collected whole buds from the two clones across the specific stages of bud burst taking into account the developmental delay between the two clones (Figure 1B, Table 1). Sustained circadian rhythmicity in DF was observed from stage II until the early part of stage IV in both clones, with posterior reduction in amplitude (dampening) during and after the stage IV (Figure 2C). Although DF was rhythmic when buds were in stage II, it showed a decreasing trend, probably due to the inability of buds to survive when detached from the tree. In addition, buds at stage II to IV showed robust rhytmicity ramping up at stage II and dampening at IV (Figure S2 available as Supplementary data at *Tree Physiology* Online). Clone 483, showed less variation than clone 142, although both displayed the same trends. Taking together these results we could provide the first evidence for the probable existence of rhythmicity studying buds in spruce, related with bud development and a very concise frame.

**Table 2:** Overview of estimated periods of DF rhythms. Data shown are means and standard errors. Rhythmic buds represent the number of buds which passed Biodare2 statistical analysis over the total number of buds analyzed.

Year	Genotype	Period (h)	SE	Rhythmic/total^*^
2022	142 week 24	13.88	1.21	2/6
	142 week 27	24.92	11.22	2/10
	483 week 27	21.51	7.62	3/10
2023	142 week 18 (dormancy)	na	na	0/10
	142 week 21	27.41	0.20	5/10
	142 week 23	12.51	0.88	5/9
	142 week 24	13.4	0	5/11
	142 week 26	33.25	8.26	5/10
	483 week 18 (dormancy)	na	na	0/10
	483 week 26	20.74	5.07	5/10
	483 week 27	19.98	5.24	5/10
	483 week 28	18.71	3.14	7/10

^*^: Significant at the 5% level. na: Not Applicable.

Going further in rhytmicity studies, we analyzed photosynthesis and respiration activities from two clones of Norway spruce. We used infra-red gas analysis (IRGA) to study photosynthetic and transpiration activities as physiological parameters for rhytmicity. This experiment was performed during 2022, and revealed a significant difference in the rate of photosynthesis (A_n_) between the two clones (Figure 2B) where samples from clone 142 had a statistically significant reduced photosynthesis level (A_n_ by clone *P*-value = 0.03842) in comparison with clone 483; other activities did not differ between clones. These results could point a genetic component which is beyond our scope.

### Analysis of gene regulatory network in Norway spruce in different light conditions

To determine the status of clock-related genes in Norway spruce, we performed a transcriptomic analysis of clone 483. Principal component analysis (PCA) of RNA-seq data from needle samples either entrained to light:dark (LD) cycles or maintained under constant light (LL) (experimental design; Figure S3A available as Supplementary data at *Tree Physiology* Online) differentiated between LD and LL for the first and second components (Figure S3B available as Supplementary data at *Tree Physiology* Online), but there was no overlap in results for the second and third components. The heatmap plot thus displayed a separation between LD and LL (Figure S2C available as Supplementary data at *Tree Physiology* Online). Both conditions were evaluated on the genes for each first level MapMan and Gene Ontology, and provided evidence for the presence of some circadian clock gene in different biological processes during LD, but of its absence under LL (Figure S3D available as Supplementary data at *Tree Physiology* Online).

Rhythmic genes under LD and LL were identified using the JTK algorithm. We identified 3952, 2078, 1899 and 1369 genes as rhythmic on LD Day 1, LD Day 2, LL Day 1 and LL Day 2, respectively (Supplementary file.xlsx available as Supplementary data at *Tree Physiology* Online). Our results were consistent with those from Ferrari et al. (2019), with respect to both the low number of rhythmic scores and the low number of shared genes (Figure S3E available as Supplementary data at *Tree Physiology* Online). None of the annotated circadian clock genes was identified as cyclic by JTK, although they were identified as such under both light conditions by Haystack.

**Figure 3:**
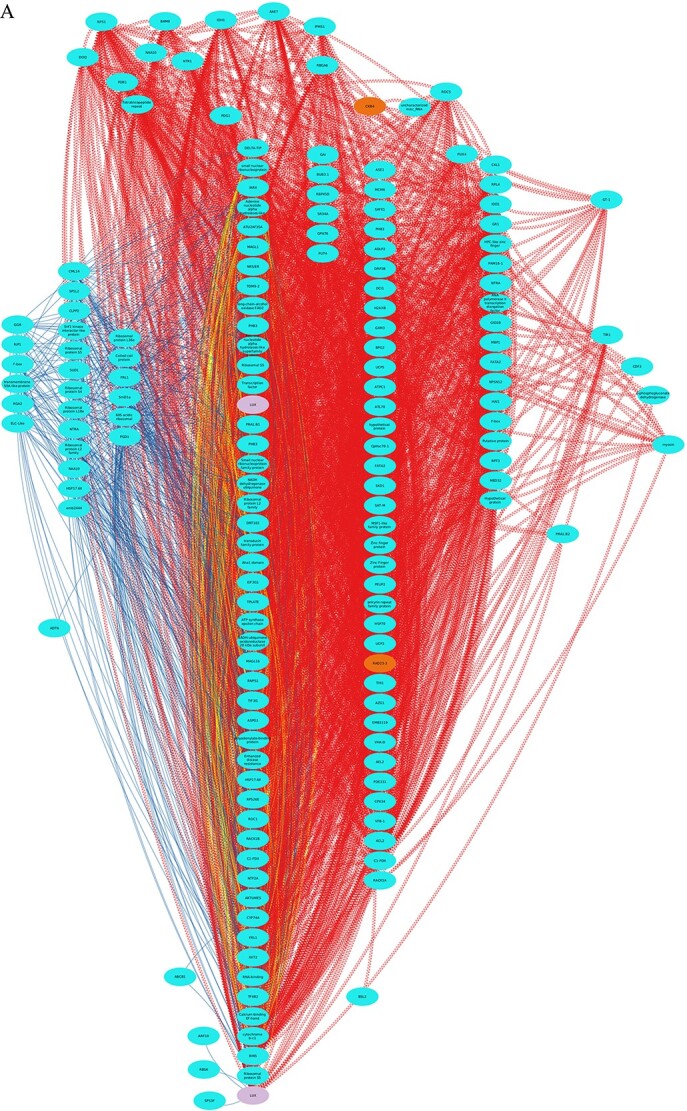

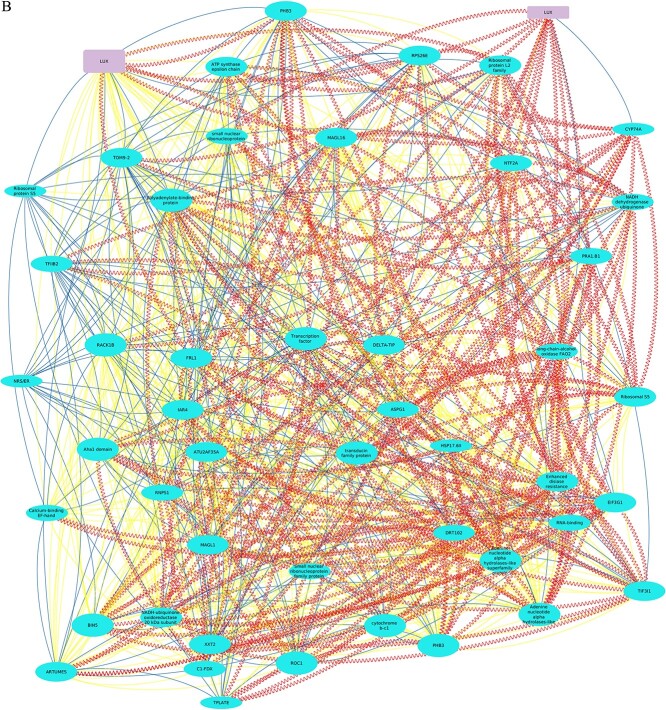

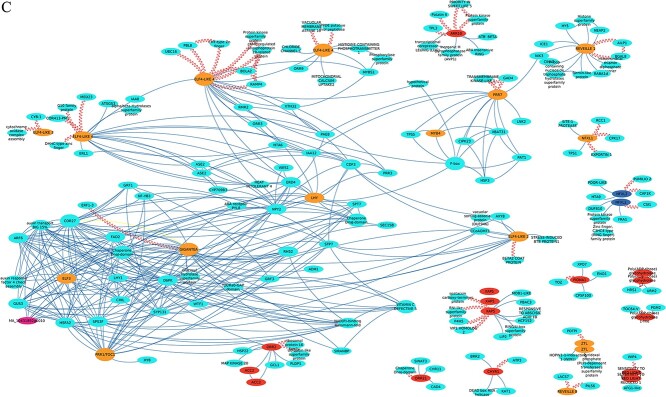
Norway spruce differentially expressed genes analysis approach under gene regulatory network. (A) Estimated gene regulatory network showing first-degree neighborhood of *LUX ARRHYTHMO* (*LUX*) based on RNA-seq data (*LUX* is shown as purple ovals in the lower part of the inserted box)*.* Red edges indicate LD; blue edges indicate LL. (B) Separate subgraph showing the central core of genes. Blue and red edges show LD and LL origins, respectively; yellow edges indicate the common, overlapping origin. (C) Estimated gene network of the first-degree neighborhood of the remaining clock genes and genes linked to them. Genes lacking definition were pruned from the network to reduce noise. Putative core clock genes are shown in orange; clock-associated genes in red; and metabolic genes in cyan. *EBI/NFXL2* is highlighted in dark blue to emphasize its importance in growth.

We wanted to go further and study possible interactions between genes depending on the light conditions. For that we performed a gene regulatory network (GRN) using Seidr (Schiffthaler et al. 2023). The network was interconnected by a number of edges discriminating between LD interactions (red color) and LL conditions (blue color). In addition we are also providing the common interactions in LD and LL in yellow (Figure 3, Figure S3F available as Supplementary data at *Tree Physiology* Online). The GRN included a main network enriched in genes related to metabolic pathways, where the gene *PCL/LUX* was linked with the core of the components generated by the network (Figure 3A). These results indicated that *PCL/LUX* was associated with the circadian clock and metabolic processes were central to the GRN (Figure 3A and B). The remaining core genes associated with the circadian clock converged, and appeared in several subnetworks, but were not accommodated within the main network (Figure 3C). These genes included *LHY*, *ELF3*, *ELF4*, *GI* and *TOC1*, as well as others associated with the clock and metabolism, such as *HYPOCOTYL ELONGATION 5* (*HY5*) and *COLD REGULATED 27* (*COR27*). Other genes related to the clock and/or metabolism were identified as interacting with one or more members of a short list of clock-associated genes *ARABIDOPSIS RESPONSE REGULATOR 10* (*ARR10*), *FIONA1* (*FIO1*), *POLY_(ADP-RIBOSE*) *GLYCOHYDROLASE 2* (*PARG2*), *REVEILLE 6* (*REV6*), *SENSITIVITY TO RED LIGHT REDUCED 1* (*SRR1*) and *ZTL* (Figure 3C). The gene models for *ACETYL-COA CARBOXYLASE 2* (*ACC2*), *CHLOROPLAST IMPORT APPARATUS 1* (*CIA1*)/*ASE2*, *XAP5 CIRCADIAN TIME KEEPER* (*XCT*), *EBI/NFLX2*, *ELF3* and *ELF4* either lacked a complete amino acid sequence or displayed duplications in their sequences and assignations.

This analysis is indicating that light in terms of day length is an important input for the clock in Norway spruce, being able to fine tune some metabolic processes in full developed needles.

## Discussion

Previous studies indicate that in 2-month-old seedlings of Norway spruce grown in greenhouse controlled conditions, provide information about the circadian clock is not active in Norway spruce seedlings at this developmental stage (Gyllenstrand et al. 2014). They concluded this by following two approaches: (i) detecting clock genes by quantitative PCR and (ii) not detecting a sustained rhythm in DF assays. In our work, we have indirect evidences of circadian clock activity by analyzing DF in buds from adult plants of Norway spruce. We observed rhythmicity during stages III and IV of buds’ development under natural conditions. Then, our data suggested that the clock is ticking in Norway spruce, and it could be influenced by geographical latitudinal gradient. The photoperiod differs geographically and it has been suggested to have a role in enabling the initiation of bud burst in Norway spruce by accumulating temperature (Partanen et al. 2001, Jönsson and Bärring 2011). Recent research points to the importance of studying circadian control mechanisms and other responses under natural conditions (Rubin et al. 2017, Oravec and Greenham 2022).

Light and temperature are important factors for the initiation of rhythmic processes coordinated with plant metabolism. In our study, we could clearly see how light is important to detect rhythmic processes during bud burst in two different clones. Norway spruce, like many other temperate trees and shrubs, shows periods of active growth and periodical rest (bud dormancy) that track the environmental conditions permitting growth in their locale. This trait is associated with activity of the circadian clock (Vince-Prue 1994, Singh et al. 2021). ‘Bud burst’ is physiologically well-characterized and has been classified into a series of defined stages (Ducci 2012). Our analysis demonstrated differences in bud burst at the clone level, both with respect to the time taken by each clone to achieve a given developmental stage and to their absolute timing; the northern clone (142) completed bud burst earlier than the southern clone (483). These results were confirmed in an independent analysis of DF in buds at different developmental stages, which demonstrated a regular frame of rhythmicity before dampening in both clones. According to the results of Gyllenstrand et al. (2014), together with Jokipii-Lukkari et al. (2018), where they detected transcriptionally, diurnal cycling of clock genes, we assume that these genes are also active during bud burst, following our DF results, providing the first evidence that the clock may be present and active in this specific time frame in conifers. This information would be crucial for further studies in clock and Norway spruce.

Many experiments are performed with models developed from annual plants such as Arabidopsis, and overall in laboratory conditions. Here, we are working with adult trees of Norway spruce and this implies several limitations in terms of techniques and information available. Our results from perennials demonstrate the requirement to consider the specific needs and biology of individual species, and the effect of temperature indicates an important relevance in the equation. In natural conditions with springtime temperatures and long day, grafted plants were not rhythmic, but summer temperatures and constant light displayed rhythmicity at studied latitudes.

We are aware of the limitations we have when doing bioinformatic analyses. However, we are providing new information about LUX interactions appearing light dependent. This could be an indicator of the presence and activity of the clock in Norway spruce. In line with this, in Jokipii-Lukkari et al. (2018) study genes we find regulated seasonally being June and July the most active months. This goes in line with our DF results and the rhytmicity found.

In our analysis of diurnal and circadian RNA-seq time-series, we demonstrated a subset of genes in Norway spruce that showed driven rhythms under LD and circadian cycles under LL. Although the genes showing rhythmic expression in needles of mature shoots identified the clock-associated gene LUX as a key player in gene expression networks, these networks were mainly related to metabolic pathways rather than the circadian clock itself. So, we have provided evidence that Norway spruce could have a functional clock and it would be more relevant during the bud burst development.

Gene expression in phloem tissues is up- and down-regulated across the seasonal cycle, with maximum activation in June and repression at the end of July, a pattern that matches the period of activity and growth in a Norway spruce clone from Hissjö (Jokipii-Lukkari et al. 2018). Their year-long study of Norway spruce growing in Hissjö, Sweden (Lat. 63.9° N; Long. 20.1° W), showed considerable seasonal variation in gene expression.

We therefore interrogated this published dataset (PRJEB9578 [ERP010702]) with the same approach of a regular RNA-seq analysis focusing on expression levels along time, and found seasonal differences in gene expression levels of putative circadian clock genes (Figure S4 available as Supplementary data at *Tree Physiology* Online), overlapping for the same period we detected rhytmicity.

Although we did only find weak rhythmic expression of a putative clock genes in mature (post-stage IV) needles, we observed a trend towards dormancy breaking in the southern clone compared with the northern clone, consistent with previous studies (Jokipii-Lukkari et al. 2018). This may result from higher levels of expression of cold-regulated genes such as *COR27*, which were identified in our network and displayed a high level of expression over the time frame of the experiment. *COR27* is associated with the circadian clock and its expression levels are under natural selection in Swedish Arabidopsis populations (Rees et al. 2021), and our results displayed relationships among *COR27* and many of the clock genes such as *GI*, *ELF3, ELF4*, *TOC1* and *LHY*. Moreover, *LUX*, which is a component of the Arabidopsis circadian clock (Hazen et al. 2005, Onai and Ishiura 2005) and also involved in temperature signaling (Chow et al. 2014), was identified as a key component at the intersection of the LD and LL transcriptomic networks. The effects of such genes, together with changing photoperiods, temperature and light intensity levels, may determine the timing of dormancy and dormancy breaking, enabling modulation of responses across accessions from different latitudinal origins. Finally, the connection between *EBI/NFXL2*, which regulates both circadian function and xylem proliferation, requires further investigation in Norway spruce, as it is likely to be instrumental in determining biomass in this species.

All in all, we are providing new insights that circadian clock through rhythmicity evidences is working during bud development. In addition, rhythmicity is strongly influenced by light intensity and LUX acts as a core in the coordination of bud burst and environmental stimuli.

## Conclusion

This study has provided evidence for the potential existence of circadian rhythms in Norway spruce. Temperature is highlighted to be a key element for future studies in bud burst compared with light conditions in the latitudes evaluated. The clock in Arabidopsis and Norway spruce could be conserved, but part of the mechanism and components in spruce could be specific, pointing the necessity of further sequencing and reassembly of the genome. The necessity to generate new models different from Arabidopsis is required in order to approach natural communities of long-lived species.

## Supplementary Material

Supplementary_Material_tpae139

Supplementary_file_tpae139

Gene_Regulatory_Network

## Data Availability

The supplementary data underlying this article are available in the article and in its online supplementary material: The DF data underlying this article are available in BioDare2 at biodare2.ed.ac.uk, and can be accessed with unique identifiers 27508, 27511, 27579, 28466, 27249 and 27254. The RNA-Seq data underlying this article are available in EMBL at https://www.ebi.ac.uk/ena/browser/home, and can be accessed with accession number PRJEB8220. The RNA-Seq data reevaluated from Jokipii-Lukkari (et al. 2018) in this article are available in EMBL at https://www.ebi.ac.uk/ena/browser/home and can be accessed with accession number PRJEB9578.
