# Supplementary material for: The circadian clock participates in seasonal growth in Norway spruce (Picea abies)
Source: Tree Physiol. 2024 Nov 3;44(11):tpae139. doi: 10.1093/treephys/tpae139 (PMC11586665; doi:10.1093/treephys/tpae139)
Supplement: Supplementary_Material_tpae139 [file supplementary_material_tpae139.pdf]

Supplementary Material

A

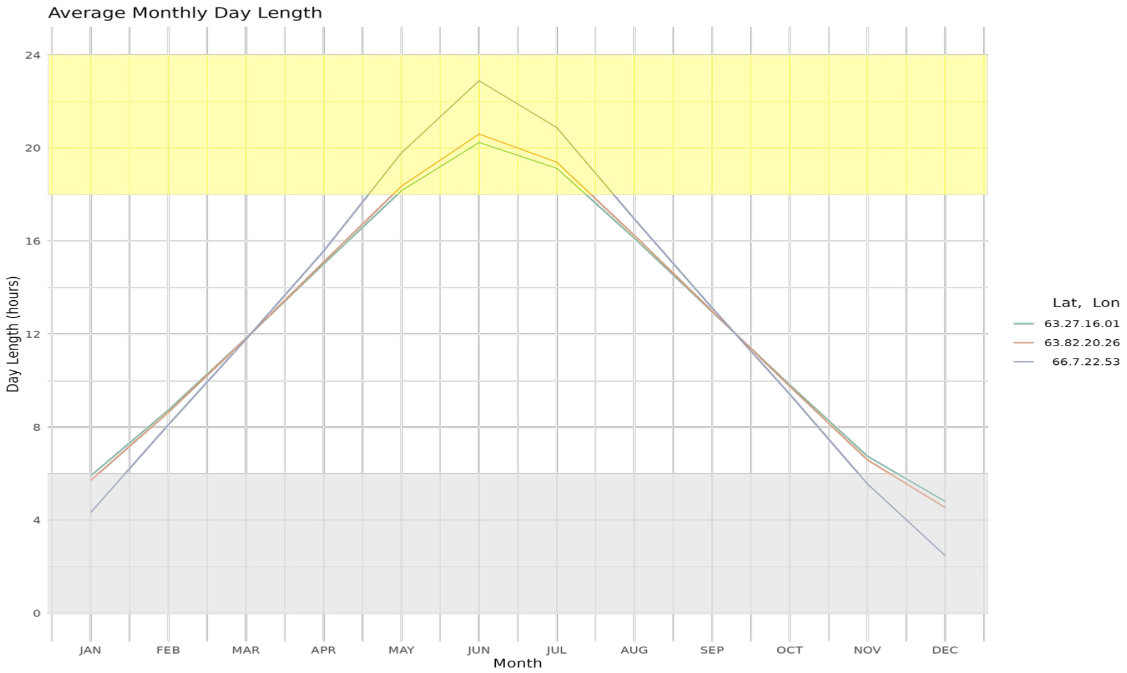

B

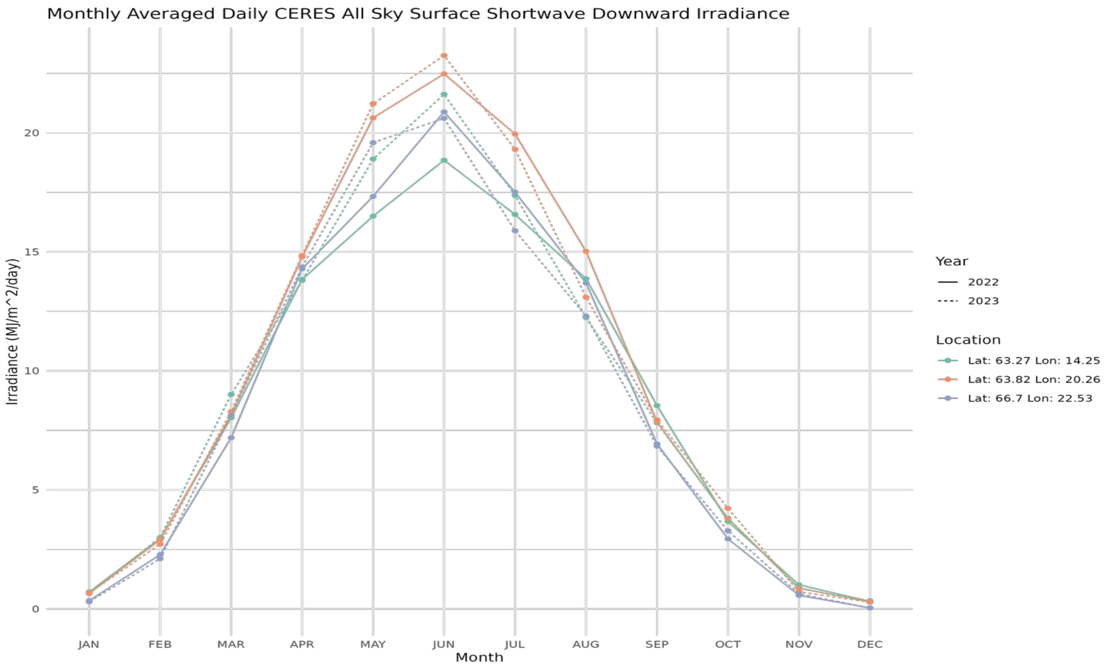

C

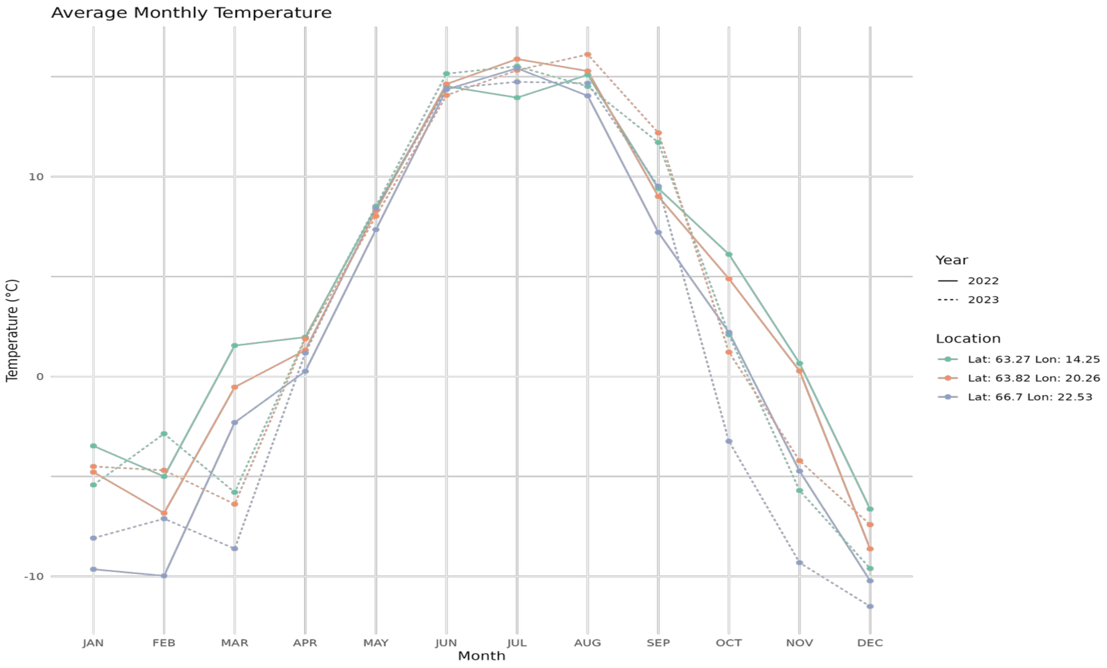

D

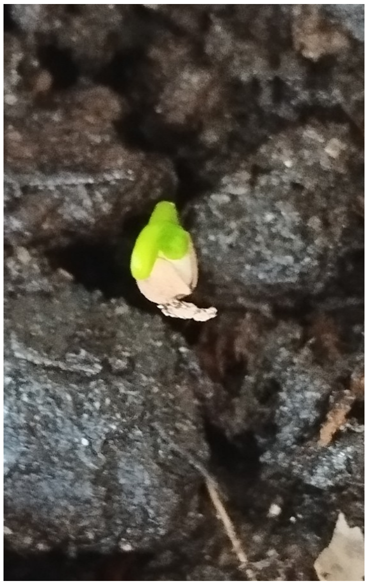

Seedling June 13th (day -5)

E

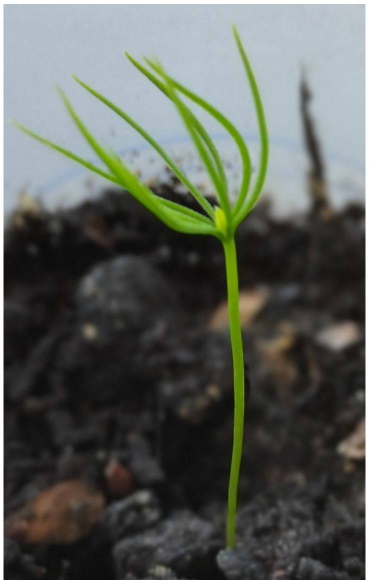

Seedling June 18th (day 1)

F

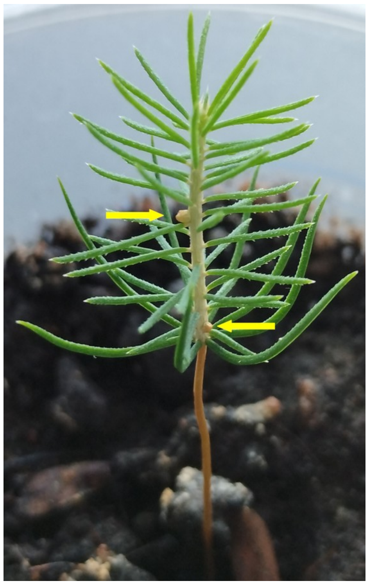

Seedling August 16th (day 59)

G

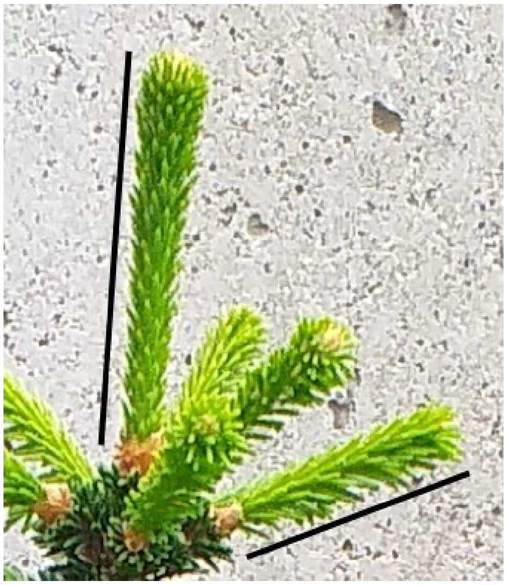

Buds on 2022, week 20 clone 142

Supplementary Figure 1. Environmental conditions registered applicable to the original location for clone 142 (66.7ºN, 22.53ºE), clone 483 (63.27ºN, 14.25ºE, and Umeå (63.82ºN 20.26ºE) as location where all experiments were carried on. **A**, average monthly day length; **B**, average Irradiance provided by NASA POWER database; **C** average monthly temperature provided by NOAA database. Day length and irradiance display the most informative parameters during the experiments in order to make inferences in the discrepancies on bud burst behavior between clones. (**D-G**) Norway spruce seedling development compared with experimental clone 142. Seed germination (D); shoot apical meristem initial development (E); two- months-old seedling with axilar buds in stem (F); buds (G)

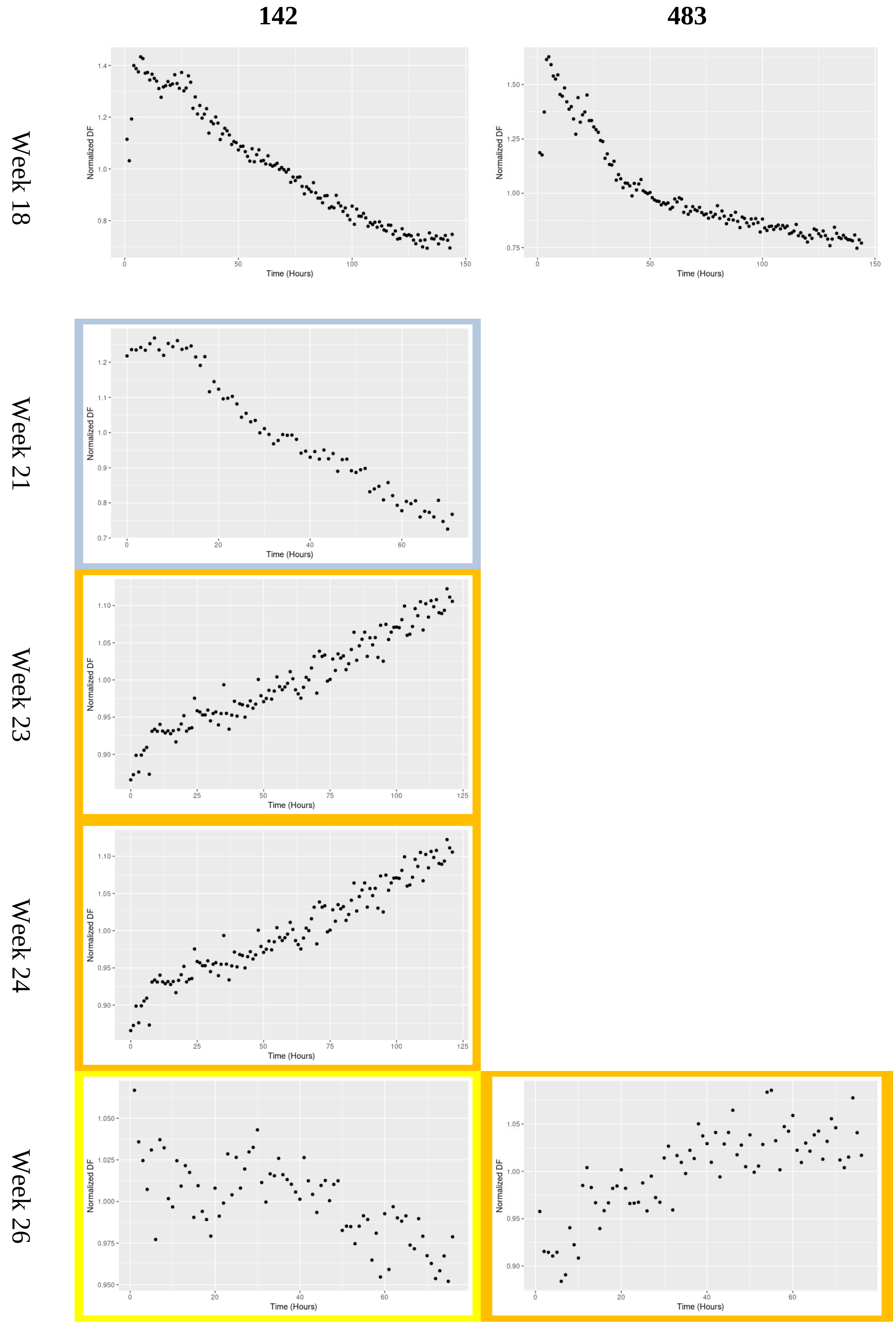

142
483
Week 18
Week 21
Week 23
Week 24
Week 26
Normalized DF
Time (Hours)

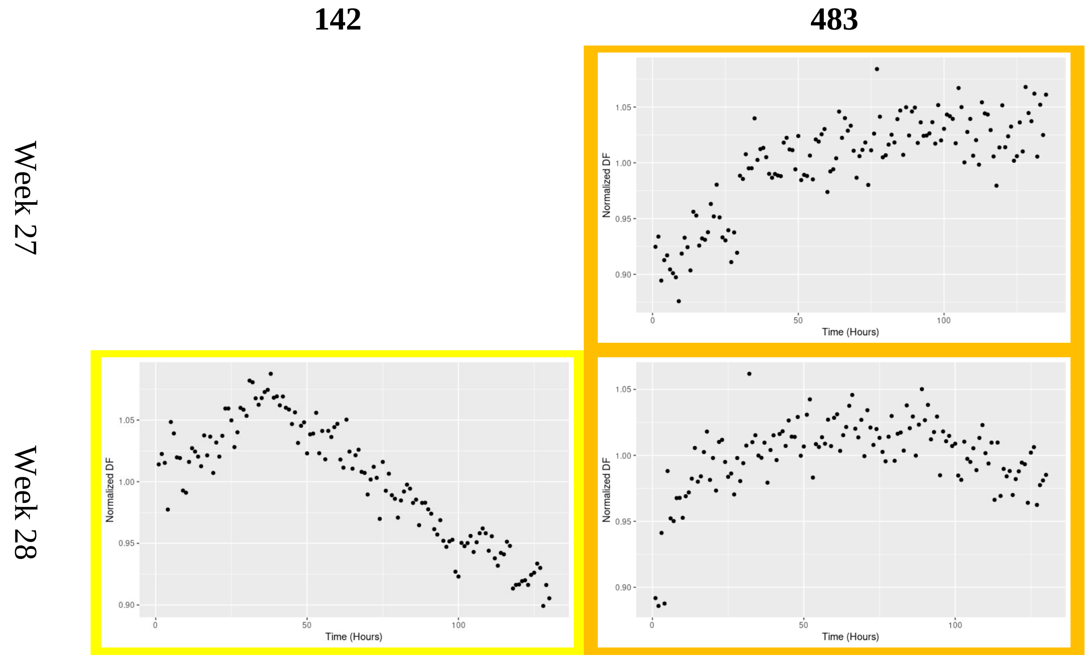

Supplementary Figure 2. Weekly raw (mean normalized) photon counts in delayed fluorescence (DF) experiments conducted in 2023 on Norway spruce buds. Box colors indicate stage of bud development. White: dormant buds; Blue: stage II; orange: stage III; yellow: stage IV. The time to transition from stage III is longer in population 483 than in population 142; buds display circadian rhythmicity at this time. Buds in stages II and IV tend to show infradian behavior with photon emissions reducing over time.

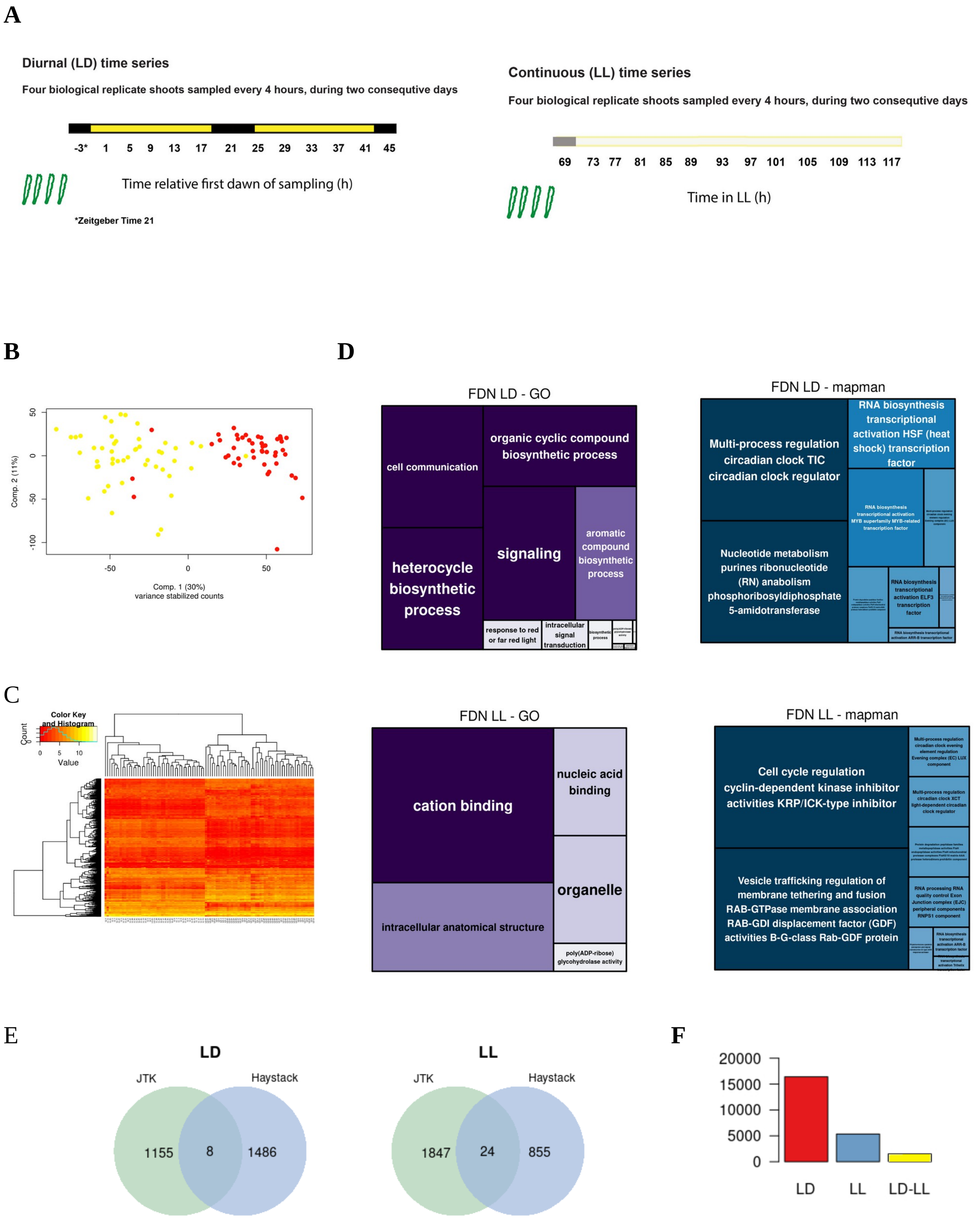

Supplementary Figure 3. Rhythmic gene expression in Norway spruce shoots under diurnal (LD) and circadian (LL) conditions. **A.** Shoots were collected randomly from four independent plants (rooted cuttings of clone 483) each timepoint under diurnal Light:Dark cycles (LD) and continuous

light (LL) conditions and used for RNA-seq experiment. **B**. Principal component analysis of RNA sequencing (RNA-seq) data from clone 483 revealed gene expression clusters representing LD (yellow) and LL (orange). **C**. Hierarchical clustering of LD and LL RNA-seq samples on the 2000 most variable genes .**D.** Enrichment plots identified from the first-degree neighborhood (FDN) output using either Gene Ontology (purple) or Mapman assignation (blue). Data shown are the results of Gene Ontology and Mapman analyses of differentially expressed genes under LD or LL. **E.** Venn diagrams showing numbers of rhythmic genes detected by Jonckheere-Terpstra-Kendall (JTK) and the Haystack algorithm in Light:Dark cycles (LD; left) and constant light (LL; right). **F.** Numbers of degree neighbors (edges) for every gene under LD (red), LL (blue) and both (yellow) from the GRN raw output.

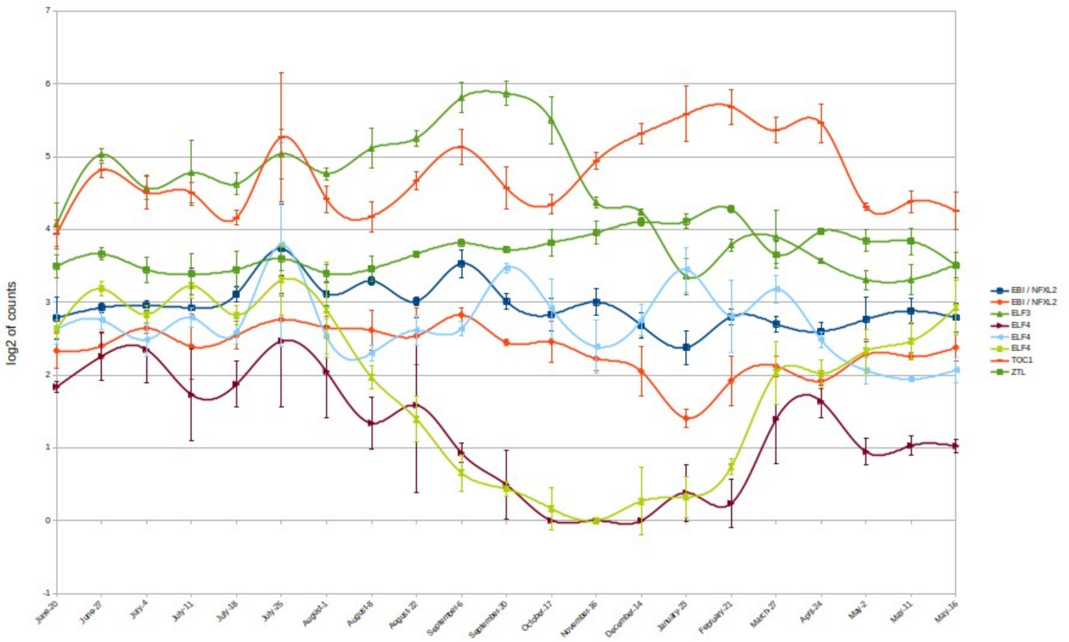

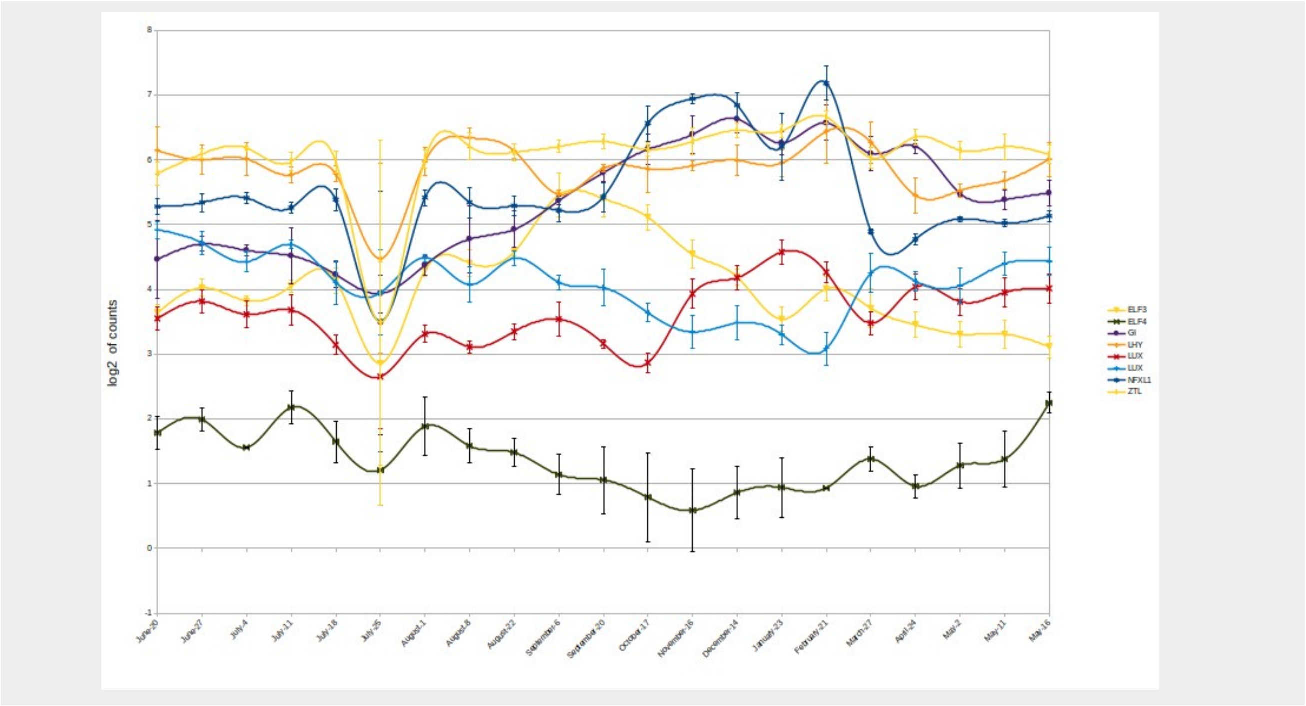

Supplementary Figure 4. Analysis of RNA-seq data from Jokipii-Lukkari et al. (2018) showing seasonal and yearly expression of putative circadian clock genes in wood tissues. Gene models were selected from the first-degree neighborhood results, individualized, and divided in two groups according to whether expression was down-regulated (upper panel) or up-regulated (lower panel) during the period of bud burst.
